# *In vivo* thyroid vibro-acoustography: a pilot study

**DOI:** 10.1186/1471-2342-13-12

**Published:** 2013-03-27

**Authors:** Azra Alizad, Matthew W Urban, John C Morris, Carl C Reading, Randall R Kinnick, James F Greenleaf, Mostafa Fatemi

**Affiliations:** 1 Department of Physiology and Biomedical Engineering, Mayo Clinic, 200 First Street SW, Rochester, MN, 55905, USA; 2 Department of Internal Medicine, Mayo Clinic, 200 First Street SW, Rochester, MN, 55905, USA; 3 Division of Endocrinology, Department of Internal Medicine, Mayo Clinic, 200 First Street SW, Rochester, MN, 55905, USA; 4 Department of Radiology, Mayo Clinic, 200 First Street SW, Rochester, MN, 55905, USA

**Keywords:** Elasticity imaging techniques, Vibro-acoustography, Thyroid neoplasm, Thyroid nodule, Ultrasound, Imaging

## Abstract

**Background:**

The purpose of this study was to evaluate the utility of a noninvasive ultrasound-based method, vibro-acoustography (VA), for thyroid imaging and determine the feasibility and challenges of VA in detecting nodules in thyroid.

**Methods:**

Our study included two parts. First, in an *in vitro* study, experiments were conducted on a number of excised thyroid specimens randomly taken from autopsy. Three types of images were acquired from most of the specimens: X-ray, B-mode ultrasound, and vibro-acoustography. The second and main part of the study includes results from performing VA and B-mode ultrasound imaging on 24 human subjects with thyroid nodules. The results were evaluated and compared qualitatively.

**Results:**

*In vitro* vibro-acoustography images displayed soft tissue structures, microcalcifications, cysts and nodules with high contrast and no speckle. In this group, all of US proven nodules and all of X-ray proven calcifications of thyroid tissues were detected by VA. *In vivo* results showed 100% of US proven calcifications and 91% of the US detected nodules were identified by VA, however, some artifacts were present in some cases.

**Conclusions:**

*In vitro* and *in vivo* VA images show promising results for delineating the detailed structure of the thyroid, finding nodules and in particular calcifications with greater clarity compare to US. Our findings suggest that, with further development, VA may be a suitable imaging modality for clinical thyroid imaging.

## Background

The clinical practice of thyroidology has been revolutionized over the last decade by the inclusion of real-time ultrasound (US) in the evaluation of nodular thyroid disease. Ultrasonography can accurately determine the number and the size of thyroid nodules. US imaging is extremely useful in guiding fine needle aspiration biopsy (FNAB); however, its role in predicting malignancy is limited such that ultrasound-guided FNAB is carried out routinely in the evaluation of thyroid nodules [1]. Conventional ultrasound characteristics of thyroid nodules are not sufficiently specific to reliably determine the malignant potential of thyroid nodules [2]. Thus, despite the obvious utility of conventional ultrasound, the gold standard for diagnosis of benign versus malignant thyroid nodules is the FNAB [2,3].

FNAB is an invasive procedure and technique accuracy depends largely on the skill of the aspirator, the expertise of the cytologist, and the difficulty in distinguishing some benign cellular adenomas from their malignant counterparts and wide variability in interpretative skill regarding cytopathology of the thyroid nodule. Because of these limitations, the results are indeterminate in approximately 15-20% of cases. Analysis of recent data from some series suggests a false-negative rate of up to 11%, a false-positive rate of up to 8%, with a sensitivity of about 80%, and a specificity of 73% [4-8].

Thyroid magnetic resonance imaging (MRI) and X-ray computed tomography (CT) scans are not as sensitive as US in detecting thyroid nodules, however, they are helpful in staging of the thyroid cancer [9,10].

Another major area of uncertainty in the management of thyroid nodules is the issue of follicular neoplasm of the thyroid. Approximately 5% of all thyroid nodules are follicular neoplasm, most of which are benign follicular adenomas, but 10-20% are follicular thyroid cancers. Neither conventional ultrasound appearance nor FNAB is sufficient to distinguish between these two lesions largely because the cytology of follicular adenoma is not sufficiently different from that of follicular carcinomas [3,11,12]. Currently, most of patients with FNAB cytology indicating “follicular neoplasm” or “suspicious for follicular neoplasm” are sent for thyroidectomy in order to make a definitive diagnosis. Thus, this issue represents a major area of clinical practice that requires new and innovative technological approaches for resolution.

Improved methods for thyroid nodule differentiation are required to effectively and appropriately select biopsy candidates in suspected nodules within the thyroid. Elasticity imaging is an emerging field of medical imaging for noninvasive and objective evaluation of tissue viscoelasticity. Magnetic resonance elastography (MRE) can be used to differentiate normal and pathological thyroid gland [13], however, this method is based on expensive MRI technology and thus less likely to see wide clinical application. Thyroid static ultrasound elastography has been developed to obtain tissue stiffness information [14,15]. The resulting image is typically the strain distribution after a stress has been applied by the user which reflects the elastic characteristics of tissues. There are very promising reports in clinical applications in thyroid nodules [16-22]. However, it has not been proven that real-time elastography alone is useful in differentiation of thyroid nodules and the technique needs additional quantitative information [23].

Acoustic radiation force impulse (ARFI) imaging and supersonic imaging (SSI) use ultrasound radiation force to generate shear waves and quantify tissue elasticity from measured propagation speed of shear wave. Studies using ARFI and SSI for thyroid nodule screening provide quantitative tissue elasticity information and preliminary results have been very promising [24-31]. However, the sensitivity of SSI and ARFI in differentiation of thyroid nodules is not high as a stand-alone diagnostic tool [32]. Thus, the role of imaging in thyroid cancer detection continues to evolve. In this paper, we present the *in vitro* and *in vivo* results of an imaging modality called vibro-acoustography on human thyroid.

Vibro-acoustography (VA) is a methodology based on the dynamic radiation force of ultrasound [33,34]. In this technique, the ultrasound energy is converted into a low-frequency vibration, which in turn produces a sound that is used to construct the image. Hence, a VA image is sensitive not only to the ultrasound properties of the object but also to the dynamic behavior of the object at low frequencies, which means that VA offers information that is not available with conventional ultrasound. We researched and evaluated the use of VA for thyroid imaging. In particular, we focused on studying images of the tissue structures and various lesions in excised thyroid samples as well as human thyroid *in vivo*. The goal of this study was to understand the properties of thyroid images and to pave the way for a larger *in vivo* thyroid imaging study by VA.

## Materials and methods

### Principles of VA

**Figure 1:**
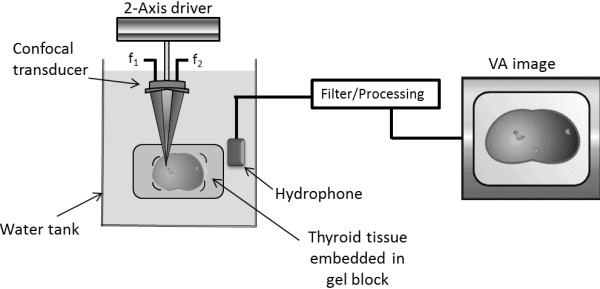
**Diagram of the experimental vibro-acoustography system for *in vitro *thyroid imaging.** The thyroid sample in the gel block is placed inside a water tank and is then scanned by the dual-beam ultrasound from the confocal transducer focused at the desired depth inside the sample. The difference frequency (Δ *f*) between the two ultrasound beams (*f*_2_ and *f*_1_) is represented as Δ *f* = *f*_2_- *f*_1_. The hydrophone receives the acoustic field at the Δ *f* from the sample. This signal is then processed and mapped into an image.

The VA technique is based on the radiation force of ultrasound, which is a nonlinear phenomenon in acoustic wave propagation [33,34]. The general principle of VA is illustrated in Figure 1.

VA uses two ultrasound beams at slightly different frequencies (*f*_1_ and *f*_2_). The ultrasound energy is transmitted in the form of two tonebursts. The beams are oriented so that they intersect at their mutual focal point. At this point, the two beams mix to produce an ultrasound field with intensity that varies at the difference frequency, Δ*f*, where, Δ*f* = *f*_2_ − *f*_1_. The ultrasound field produces a radiation force [33,34] on the object at the focus of the combined beams. This force is proportional to the intensity of the ultrasound field; hence, it varies in time at the Δ*f*. As a result, a sound field at the Δ*f* is produced and is detected by the hydrophone. The hydrophone signal represents the object’s dynamic response to the force exerted at one point in the object. The hydrophone signal is used to modulate the brightness of a point on the display that corresponds to the position of the focal point on the object. As the ultrasound beams are scanned across the object, the VA image of the object is constructed on the display. Although the image is constructed from a low-frequency signal at Δ*f*, which is often in the kilohertz range, the spatial resolution of the image is determined by the focal spot size, which is typically in submillimeter to millimeter range—a range very suitable for clinical imaging. Figure 1 demonstrates a diagram of the experimental vibro-acoustography system for thyroid imaging.

We note that VA images are in the C-plane, i.e., the plane perpendicular to the ultrasound beam, which is also perpendicular to the B-plane used in B-mode ultrasound imaging. This C-plane is a constant distance from the surface of the transducer. Therefore, VA and B-mode present two perpendicular cross-sections of tissue.

A VA image depicts two types of information about the object. First, the image represents the ultrasonic properties of the object, such as its scattering and power absorption characteristics. These properties are also present in traditional ultrasonography [35]. The second type of information is the dynamic characteristics of the object at the Δ*f*, which describes how the object responds to a vibrating force at low frequencies [35]. This information is not available from conventional ultrasound imaging. Because VA images are formed using the acoustic signal at a low frequency, the resulting images are speckle free, which is an important advantage over traditional ultrasound imaging [36].

VA has been tested in numerous potential clinical applications, including *in vitro* experiments on heart valves [37], *in vitro* human vessels [38], *in vivo* animal arteries [39], breast tissue [40,41], liver tissue [42], bone [43], and, more recently, *in vivo* human breast [44-46]. Recent reports on prostate applications of VA include evaluation of VA for imaging prostate brachytherapy seeds [47] and for monitoring prostate cryotherapy [48]. It should be noted that VA in its present form is not intended for quantitative imaging. Diagnostic information from VA is based on image contrast, which is also the case in most other imaging modalities.

### Imaging in vitro thyroid tissues

In an *in vitro* study, after obtaining the approval of the Mayo Clinic Institutional Review Board (IRB) for tissue specimens, experiments were conducted on a number of whole excised human thyroids from autopsy. The specimens were randomly taken and there was no previous knowledge of the presence of nodules inside these thyroid tissues. Tissue samples were fixed in 10% formaldehyde for 1 hour to prevent potential contamination, after which they were thoroughly rinsed in water to remove residual formaldehyde before embedding [42]. Tissues were embedded in a tissue mimicking gel block (~10 x 10 x 3 cm). To make the gel block, gelatin powder (G2500, Sigma-Aldrich, St. Louis, MO) was first dissolved in water in a 60 g/L ratio and heated to 50°C. Once the gelatin was fully dissolved, glycerol (G7757, Sigma-Aldrich, St. Louis, MO) was added at 10% by volume. The solution was then cooled to 42°C and the tissue specimen was placed in the liquid gel. The tissue and liquid were degassed under vacuum to remove air from both the tissue and liquid. After degassing, the liquid and tissue specimen were carefully poured into a square mold and refrigerated overnight before scanning.

**Figure 2:**
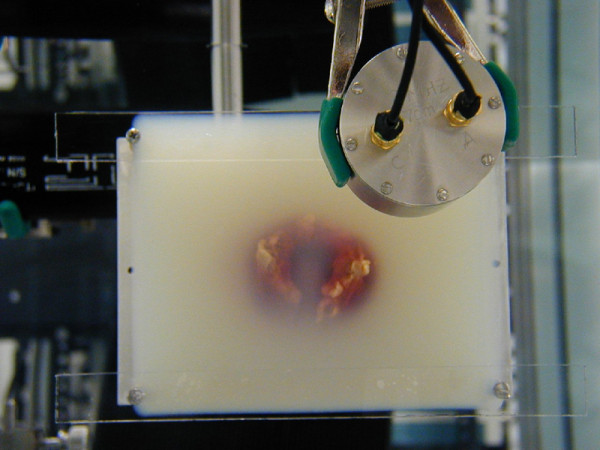
**Experimental setup for *in vitro *thyroid imaging by vibro-acoustography.** The excised thyroid is embedded in a gel block.

The VA experiment was conducted in a water tank; the diagram of the experimental VA for *in vitro* imaging is shown in Figure 1, where the tissue is scanned by a dual-beam confocal ultrasound transducer focused at the desired depth inside the sample. VA images were acquired from each specimen. VA images, 50 × 50 mm, were acquired at several Δ*f* frequencies normally ranging over 30–90 kHz. In addition to performing VA with tonebursts of length 100 μs, we also used two intersecting continuous wave (CW) focused ultrasound beams of different frequencies to generate a localized oscillatory stress field for imaging. Figure 2 shows a photograph of an excised thyroid embedded in a gel block being scanned by a dual-beam confocal transducer. Specimens were also imaged with a high-resolution specimen radiography machine (M50; Faxitron, Wheeling, IL), dedicated for research.

### Imaging human thyroid in vivo

After obtaining IRB approval, we recruited 24 (10 male and 14 female) patient volunteers, who were being evaluated for thyroid nodularity and were referred to the Department of Radiology for FNAB. Ages of the subjects ranged from 37 to 82 years old with a mean of 57.8 years old. Normally, these patients have already undergone diagnostic ultrasound examinations. Because the biopsy needle may alter tissue characteristics, VA was performed before FNAB to avoid these possible errors. B-mode ultrasound and VA images of thyroid were obtained from 20 patients. The patient had his/her clinical FNAB after this step as part of his or her health care. The nodule or nodules selected for FNAs were marked as a region of interest on thyroid ultrasound by our radiologist (C.C.R.), which later was used for VA nodule detection. The results of biopsy were analyzed and evaluated by a pathologist.

**Figure 3:**
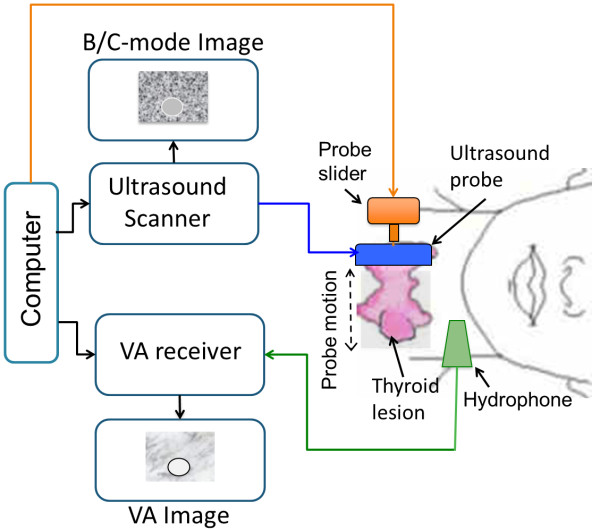
**Diagram of the dual modality system for *in vivo *VA and B-mode imaging.** The system can produce both B-mode and VA images. The computer-controlled probe slider translates the probe across the region of interest (thyroid). A collection of B-mode images is recorded as the probe slides across the thyroid. The same sliding motion is used to acquire a VA image of the same region. The C-mode image is extracted from the B-mode data at a depth that corresponds to the VA image plane.

The diagram of the dual modality system for *in vivo* VA and B-mode imaging is shown in Figure 3. For B/C-mode and VA imaging we used a modified General Electric ultrasound scanner (Vivid 7, GE Healthcare Ultrasound Cardiology, Horton, Norway), which was described by Urban, *et al.*[49] A linear array transducer (7 L, GE Healthcare Ultrasound Cardiology, Horton, Norway) was used to scan the thyroid.

The transducer was mounted on a linear translation stage that was controlled by an external PC. The transducer moved in the elevation direction of the transducer to acquire B-mode images at consecutive parallel planes; also the same mechanism is used to acquire VA data from consecutive parallel lines of the VA image. The C-mode images are formed by summing the backscattered ultrasound signal from the B-mode images using a window that was centered at a specified depth and had a length of 2.5 mm.

For VA, the scanner produced two ultrasound beams with different frequencies both focused at a joint focal point. Electronic focusing delays are used to focus at different focal depths. The two beams were electronically steered together across the object along the length of the array (also called azimuthal direction). Comparing to the system used for *in vitro* experiments, the steering function of the array probe along the azimuthal direction replaces the similar scanning motion that was performed for the *in vitro* VA imaging by mechanical means. The ultrasound frequencies were around 3 MHz and Δ*f* was around 50 kHz. In most cases, the ultrasound beams had frequencies *f*_1_ = 3.64 MHz and *f*_2_ = 3.58 MHz making Δ*f* = 54 kHz. The beams were constructed using an aperture with 64 elements which were assigned a signal with frequency *f*_1_ adjacent to a group of 64 elements, which were assigned a signal with frequency *f*_2_. This aperture was translated across the transducer array as described by Urban, *et al.*[49].

The hydrophone was placed on the neck of the subject and the acoustic emission signal was recorded during the VA scanning procedure. Multiple planes in depth were investigated during each scanning session. All the VA images were 50 × 47 mm.

## Results

### *In vitro* study results

**Figure 4:**
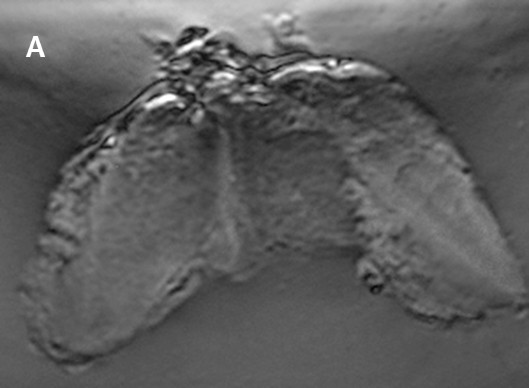
**VA image of an excised human healthy thyroid tissue.** The image size is 55 × 75 mm. The image is taken at 6 mm depth and shows normal thyroid tissue structure with no nodule and calcifications.

**Figure 5:**
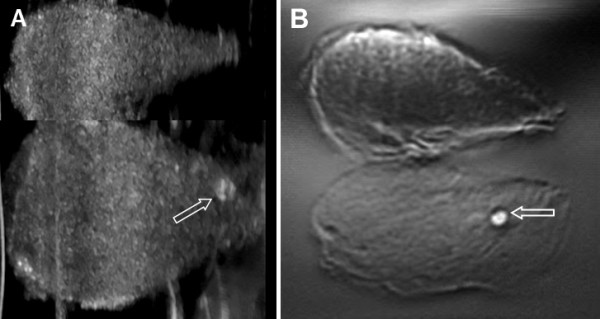
**Excised Human Thyroid. A**) C-mode ultrasound of an excised human thyroid from a cadaver, **B**) VA image of this tissue in 60 × 60 mm scan dimension. The arrow shows a 2.5 x 2.5 mm calcification in VA image with more contrast than shown in the US image.

To optimize the VA system for *in vivo* human studies, we initially tested the system on 20 excised human whole thyroid tissues. Vibro-acoustography images of excised thyroids displayed calcifications and nodules. Thyroid nodules and cysts were detectable. Image contrast was significantly higher than ultrasound images. The VA appearance of a normal excised human thyroid tissue with no nodules is seen in Figure 4. In Figure 5, a large calcification, about 2.5 mm in diameter, is seen in VA and US images of an excised human thyroid from a cadaver.

**Figure 6:**
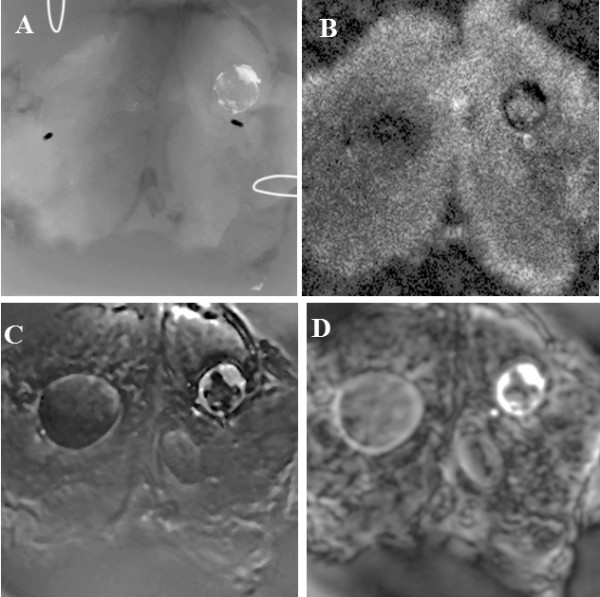
**Excised Human Thyroid. A**) X-ray of an excised thyroid tissue from cadaver; **B**) US C-scan of the same tissue at 5 MHz; **C**) VA image using toneburst excitation with Δ*f* = 50 kHz. The image is 50 × 50 mm. **D**) VA image using CW excitation with Δ*f* = 16.3 kHz. VA images were taken in water tank. All VA images are 50 × 50 mm.

X-ray, C-mode US and VA images of an excised thyroid tissue are shown in Figure 6. Three lesions, one of which is calcified, are seen in all VA images. In the X-ray image only the calcified lesion is seen, but the other two are not visible. In the C-mode ultrasound image (tissue was cut in 2 pieces) the nodules can be seen. In general, image contrast in the VA images was substantially higher than that in the ultrasound images in VA tissue experiments. In the *in vitro* study, we assessed the appearance of calcifications (CAs) and nodules in vibro-acoustography by classifying each image into one of these three categories: (A) Positive (clearly identified calcifications at locations indicated in the corresponding X-ray image or nodules at the locations indicated in the corresponding C-mode US); (B) Suspicious (image showed some indication of calcifications that could not be clearly differentiated from the background tissue and in case of nodules the border was not clearly defined); and (C) Negative (no calcification could be distinguished at locations indicated in the corresponding X-ray image and no nodules could be distinguished at locations indicated in the corresponding C-mode US image).

VA was able to detect all (3/3) of X-ray proven calcifications and all (2/2) of ultrasound proven nodules within thyroid tissues (Table 1). The measurements of the top right calcified nodule were 8.8 mm × 7.9 mm in X-ray; 7.9 mm × 7.2 mm in C-mode; and 10.3 mm × 8.7 mm in VA images. Since the goal of this *in vitro* study was to optimize the system for our *in vivo* imaging, tissue samples were randomly chosen from human cadavers regardless of pathology.

**Table 1:** **Summary of VA experimental results on 20 *ex vivo *thyroid samples**

	**Positive**	**Suspicious**	**Negative**
Presence of X-ray proven calcification in VA	3/3	0	0
Presence of ultrasound proven Nodules in VA	2/2	0	0

### *In vivo* study results

**Figure 7:**
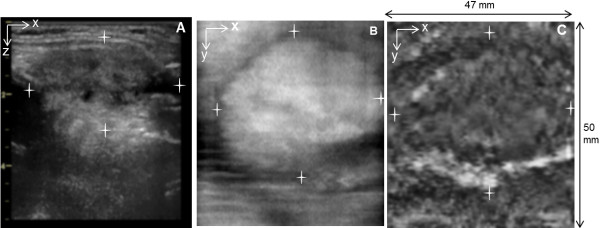
**Benign Thyroid Nodule. A**) B-mode US of a patient’s thyroid shows a large solid nodule measuring 47×27 mm in the *x***-***z* dimensions on left lobe with a small cystic component, **B**) Ultrasound C-scan at 2.0 cm depth measuring about 44.2 mm x 30.5 mm in the x-y dimensions, and **C**) *In vivo* VA image of thyroid (with 7 L array probe, *f*_1_ = 3.64 MHz and Δ*f* = 54.27 kHz) at 2.0 cm depth which correlates to the one of C-scan plane, measuring more than 47 mm in the *x*-direction and 30.5 mm in the *y*-direction and the star shows the dimensions. The VA image is 47 ×50 mm.

**Figure 8:**
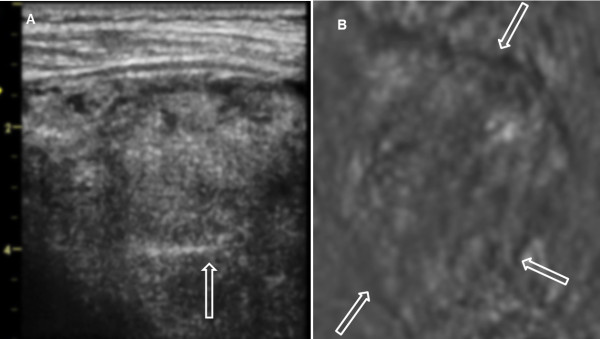
**Benign Thyroid Nodule with Degenerative Changes. A**) B-mode US of 71 year old patient’s thyroid on left side. The arrow points to a nodule measuring 2.9 × 4.2 × 4.0 cm, **B**) VA image at 2.25 cm depth, the arrows point a large nodule covering most of image. The pathology result was a benign nodule with degenerative changes. The VA image is 47 × 50 mm. Orientations of the images are similar to those as Figure 7.

**Figure 9:**
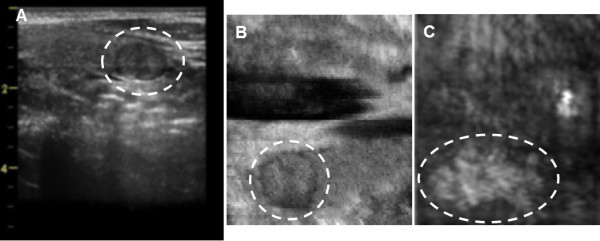
**Benign Thyroid Nodule. A**) B-mode US of left side of patient’s thyroid identifies a nodule measuring 1.4 × 1.4 cm isoechoic nodule protruding from the lower pole of the left thyroid lobe, **B**) C-mode US at 2.25 cm depth shows the nodule, **C**) VA scan at 2.25 cm depth shows nodule at the same location as seen in C-mode but larger, measuring about 1.0 × 1.5 × 3.4 cm. Orientations of the images are similar to those as Figure 7.

**Figure 10:**
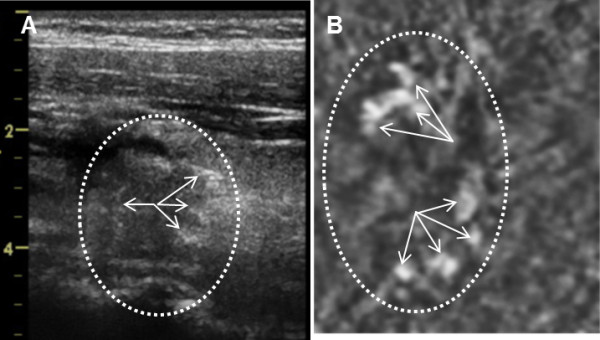
**Papillary Thyroid Cancer. A**) B-mode US shows a cystic nodule measuring l.0 × l.3 × l.4 cm in the upper right lobe of the thyroid with peripheral calcifications as denoted by arrows. VA scan at 2.5 cm depth shows the nodule with cystic appearance slightly larger dimensions plus some coarse peripheral calcifications. Orientations of the images are similar to those as Figure 7.

**Figure 11:**
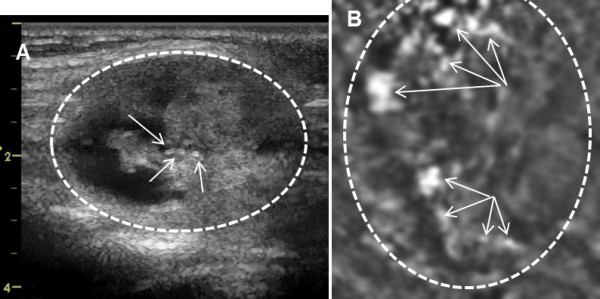
**Papillary Thyroid Cancer. A**) B-mode US shows a cystic nodule measuring 2.6 cm × 2.9 cm × 3.7 cm and a solid nodule about 2.1 cm in greatest dimension with peripheral coarse calcifications in the inferior left lobe. **B**) VA scan with array probe at 2.0 cm depth shows the ill-defined nodules, and partial cystic appearance. The VA scan is taken at the largest diameter of nodule of shown at B-mode and appears larger covering most of the image, (shown by circle) with arrows pointing to the calcifications. Orientations of the images are similar to those as Figure 7.

**Figure 12:**
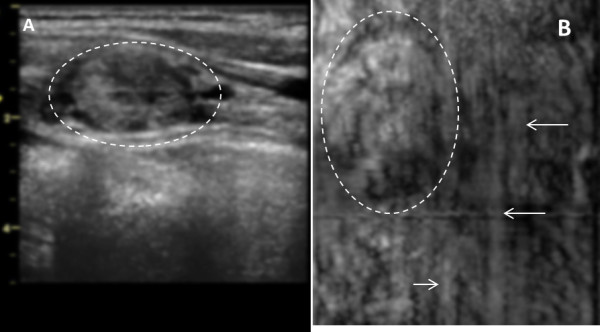
**Benign Thyroid Nodule with Degenerative Changes.** (**A**) B-mode US of left lobe of a patient’s thyroid, circle points to an oval nodule measures 2.0 cm × 1.5 cm × 1.5 cm and (**B**) VA image at 2 cm depth (with 7 L array probe, *f*_1_ = 3.64 MHz and Δ*f* = 54.27 kHz ) show a large nodule in a human thyroid marked by circle. The vertical bands marked by the arrows are system artifacts. The pathology analysis revealed that this was a benign nodule. Orientations of the images are similar to those as Figure 7.

We performed thyroid VA and US imaging on 24 adult patients on one side and on two lobes in one patient. All the VA scans were 50 × 47 mm. The results from six patients are shown in Figures 7, 8, 9, 10, 11, 12.

Ultrasound data are depicted in B-mode and C-mode. C-mode image plane corresponds to VA image plane, where this plane is perpendicular to the B-mode image plane. To clarify image orientation, we use a three-axis Cartesian coordinate system of *x*, *y*, and *z*, where *x* represents the scan direction in B-mode image (also known as azimuth direction), *y* represents the probe motion (also known as elevation direction), and *z* representing the depth. Ultrasound B-mode images are presented in the *x*-*z* plane, while C-mode and VA images are in the *x*-*y* plane.

Figure 7 demonstrates US and VA images of the left thyroid lobe of a 48-year-old female patient with a benign thyroid nodule. B-mode ultrasound identifies a large nodule measuring about 47 × 27 mm (*x*-*z* dimensions) with small cystic appearance. The nodule appears on C-mode US, taken at 2 cm depth, measuring about 44.2 mm × 30.5 mm (*x*-*y* dimensions). The VA image taken at 2.0 cm depth identifies the large nodule with distinct margin measuring about 30.5 mm in the *y*-direction and more than 47 mm in the *x*-direction, with its side margins extending out of VA image window. Its shape and location correlates to the US C-scan at 2 cm depth. Note that the resolution cell of the VA system in the depth direction was around 2–3 mm, which is similar to that of C-mode images.

Figure 8 demonstrates US and VA images of a 71 year old man with a nodule on the left side of his thyroid. The nodule on B-mode US measures about 29 × 42 × 40 mm. On the VA scan at 1.75 cm depth, the nodule appears larger and almost covers most of the scan image with little normal tissue around, as denoted by arrows. It should be noted that the VA scan depth was set to be where the largest dimension of the nodule was observed in the B-mode US image. The histology of the nodule revealed benign appearance with degenerative changes.

Figure 9 demonstrates US and VA images of the left side of the thyroid of a 56 year old female with a benign adenoma. The nodule appears on B-mode and C-mode US, taken at a depth of 1.25 cm, as an isoechoic nodule protruding from the lower pole of the left thyroid lobe measuring 12.5 mm × 12.0 mm in C-mode. The VA scan at 2.25 cm depth identifies the nodule at the same location seen in C-mode US but larger, measuring about 11.6 × 23.8 mm.

Figure 10 demonstrates US and VA images of the right side thyroid of 37 year old male with papillary thyroid carcinoma. On B-mode US, a cystic nodule appears measuring about l.0 × l.3 × l.4 cm in the upper right lobe of the thyroid with peripheral calcifications, characteristic of a papillary carcinoma. The VA scan at 2.5 cm depth identifies the nodule with a cystic appearance and slightly larger dimensions plus some peripheral calcifications, denoted by arrows, with greater clarity than shown in the US image. It is difficult to measure the size of the nodule in both the US and VA images reliably due to indistinct margins.

Figure 11 presents US and VA images of a 41-year-old woman with a thyroid nodule in the left lobe. On B-mode US, two nodules appear, a cystic nodule measuring 2.6 × 2.9 × 3.7 cm and an almost solid nodule about 2.1 cm in greatest dimension with the coarse calcifications in the inferior left lobe. The VA scan with the array probe at 2 cm depth identifies the ill-defined nodules denoted by the circle. The VA scan is taken at the largest dimension of nodule of its B-mode image. For this reason, the nodule appears larger and covered most of the image. Coarse calcifications appear on the VA image with greater clarity as shown by arrows. The VA and US appearance were suggestive for malignancy and the pathology of FNA sample from the inferior left thyroid nodule containing coarse calcifications revealed papillary thyroid carcinoma.

Figure 12 demonstrates US and VA images of the left side thyroid of an 82-year-old male patient with benign thyroid nodule with degenerative changes. In the inferior aspect of the left thyroid lobe there is an oval well defined heterogeneously echogenic nodule that measures 2.0 × 1.5 × 1.5 cm on B-mode ultrasound. The VA scan at 2.0 cm depth identifies the nodule slightly larger, as denoted by circle.

To assess the appearance of clinical ultrasound proven nodules and CAs in B-mode and or C- mode, we classified each B/ C-mode image into one of the following categories: A) Good (nodules and CAs were clearly seen at locations indicated in the corresponding clinical US, B) Fair (image showed some indication of calcifications that could not be clearly differentiated from the background tissue and in case of nodules part of the border could not be identified. C) Inconclusive (no calcification and nodules could be distinguished at locations indicated in the corresponding clinical US). The same classification criteria were used for presence of CAs and nodules in VA images were compared to the corresponding B/C mode US.

In summary, as shown in Table 2, VA was able to detect 20 nodules out of 22 nodules that were detected in the corresponding B/C-mode images, where 12 images were classified as Good and 8 were classified as Fair, because VA images were affected by some types of artifacts but the lesion was still detectable. In 2 cases the VA image was classified as inconclusive.

**Table 2:** **Results summary of 24 *in vivo *thyroid VA experiments**

	**Detctable**	**Inconclusive**
Appearance of nodules by B-mode	22/24	2/24
Appearance of B-mode US proven nodules in VA	20/22	2/22
Appearance of Calcifications on B-mode	2	4/6
Appearance of Calcifications in VA	6	0/6
		
	**Malignant PTC**	**Benign nodules**
Histology of 20 VA detected nodules	2	18

VA was able to detect the US-detected calcifications (CAs) with greater clarity though they appeared larger than in US. In a number of experiments, some levels of either system artifact or body motion artifact, mainly due to breathing, were present. In two cases the artifacts were dominant and masked the nodules. The pathology revealed papillary thyroid carcinoma in two cases of VA-detected nodules and the rest were benign. The two papillary carcinomas had pathology-proven calcifications that could be seen in US as well as VA images.

## Discussion

In the *in vitro* study, the VA images could visualize normal tissue structures and lesions free of the speckle associated with conventional B-mode ultrasound as demonstrated in Figures 4, 5 and 6. Figure 6 demonstrates that the X-ray was only sensitive to one of the lesions that were present and US revealed two nodules with no apparent calcifications, whereas the VA images could detect all three nodules and calcifications in the thyroid tissue. Additionally, the contrast and the edge detail were enhanced in the VA images as compared to the US images. It should be noted that, VA in the present form is not intended as a quantitative imaging. Similar to most other medical imaging modalities, diagnostic information in VA is based on image contrast.

It is important to note that formaldehyde can significantly change tissue properties; particularly it increases tissue stiffness. However, we also note that the study on excised tissue will have the benefit of providing valuable information and allowing us to optimize our system before moving to *in vivo* studies. Although short term fixing with formaldehyde somewhat alters the stiffness of the thyroid tissue, such changes affect both healthy and diseased areas. Hence, tissue fixation should not have a major effect on the capability of VA imaging to detect a contrast between normal and diseased tissue [42].

The confocal transducer used in this study was mechanically scanned in a water tank for the *in vitro* portion of this study. With this experimental setup the scan time could take 6–8 minutes. In order to translate this method for clinical use and improve the scan times, the modified scanner equipped with the linear array was used for the *in vivo* study. This system acquires images of similar size in about 2 minutes. It should be noted since the beamforming technique used for *in vivo* imaging is significantly different from that used for in vitro imaging; the resulting image quality is not the same. One reason for such difference is the fact that the confocal transducer used in the *in vitro* experiments has a large circular aperture that is capable of producing high-resolution images with symmetric resolution cells. Such images are typically smooth and clear. The beamforming with linear array used for the *in vivo* study has a much smaller aperture thus resulting in lower resolution images and non-symmetric resolution cell. This is a limitation of the ultrasound scanner – not the VA technique – because the ultrasound scanner can only support linear (rectangular) arrays with relatively small apertures.

The results from the *in vivo* study show that nodules could be detected by in the VA images and the edges could be defined. Size of nodules appeared larger in most of VA images than in the corresponding B-mode and C-mode images. The difference may be attributed to the fact that the information content of VA and B-mode are different; that is, VA is sensitive to properties of tissue at ultrasound as well as low (Δ*f*) frequencies, whereas B-mode and C-mode images are sensitive only to the ultrasound properties of tissue. VA was able to detect all the calcifications, in the form of micro- and coarse calcifications, within the nodules with greater clarity than those of seen in US. The histology of nodules proved the presence of calcifications including in two papillary carcinomas. It is known that the presence of calcification within solitary thyroid nodule is highly associated with thyroid malignancy [50-54]. VA is sensitive to calcifications and to our experience [37,41,45,46], it shows calcifications better than ultrasound. In US calcifications are often detected by their shadow, but in VA we can directly see and identify calcifications.

There were a few difficulties that were encountered in the *in vivo* study. First, we have identified a system artifact associated with steering the VA beams across the aperture which creates streaks in the resulting image. These types of artifacts were observed in Figure 12B as marked by arrows. We have developed an algorithm to correct for these streaks [49], but this algorithm can, in some instances, diminish image details and we are working on alternatives to correct this artifact.

Additionally, during the *in vivo* scanning, there is an artifact associated with motion due to patient breathing. This breathing artifact is manifested as jagged image details. A mild case of this artifact is shown in Figure 7C with jagged edges in the horizontal direction when comparing adjacent vertical rows. When the patient breathes, the thyroid can move in and out of the VA focal plane, so the same tissue is not necessarily excited. Because the scan takes about 2 minutes, we could not ask the subject to simply hold their breath. We did ask the patient to breath shallowly to avoid large excursions. We also placed the transducer on a spring mounted assembly so that the transducer would move with the patient during breathing as well as conform to the neck of the subject. Another limitation of this study is the limited sample size. It should be noted that the goal of this paper is to report the results of a pilot study conducted on a small number of thyroids and provide the basis for future studies. We did not intend or claim to have sufficient number of samples for a complete statistical analysis. For this reason we have not presented a sample size calculation. Results of this study may be used to determine the sample size for future studies. A larger study would provide a more accurate assessment of the sensitivity and specificity of thyroid nodule detection by VA. Results of this paper suggest that VA may be used as an additional thyroid imaging tool, however, this method is not meant to replace B-mode US or FNAB at present time.

## Conclusion

Herein, we have reported on our examination of the use of VA for *in vitro* and *in vivo* imaging of the thyroid. This study was a prerequisite for a larger population of thyroid patients. Our experiments on excised and *in vivo* thyroids demonstrate the capability of VA for the detection of thyroid nodules and calcifications. These results suggest that VA may be a useful clinical tool in thyroid imaging; however, at present VA is not intended to replace B-mode US or FNAB. In conclusion, our pilot study has shown that thyroid vibro-acoustography is feasible. However, larger studies are needed to determine sensitivity and specificity of this imaging tool in thyroid cancer detection and differentiation.

### Consent section

Written informed consent was obtained from the patient(s) for publication of this manuscript and accompanying images.

## Abbreviations

f1: frequency 1; f2: frequency 2; VA: Vibro-acoustography; Δf: Difference frequency; MRI: Magnetic resonance imaging/image; US: Ultrasonographic, ultrasonography, ultrasound; VA: Vibro-acoustography; PTC: Papillary thyroid carcinoma.

## Competing interests

Disclosure: Drs Greenleaf, Fatemi, and Alizad disclose Mayo Clinic’s patents on the vibro-acoustography technology (discussed in this manuscript) as a potential financial conflict of interest.

## Authors’ contributions

AA: conducting human study, writing most of the manuscript. MWU: image processing, writing some of discussion section, manuscript editing. JCM: patient selection, image interpretation, manuscript editing. CCR: patient selection, image interpretation manuscript editing, RRK: system technician, operating the vibro-acoustography system, some of method section. JFG: technique development, vibro-acoustography system design, manuscript editing. MF: technique development, vibro-acoustography system design, writing the technical section of the paper, supervising data acquisition and signal processing. All authors read and approved the final manuscript.

## Pre-publication history

The pre-publication history for this paper can be accessed here:

http://www.biomedcentral.com/1471-2342/13/12/prepub
